# The mechanism and active compounds of semen *armeniacae amarum* treating coronavirus disease 2019 based on network pharmacology and molecular docking

**DOI:** 10.29219/fnr.v65.5623

**Published:** 2021-02-04

**Authors:** Yuehua Wang, Wenwen Gu, Fuguang Kui, Fan Gao, Yuji Niu, Wenwen Li, Yaru Zhang, Zhenzhen Guo, Gangjun Du

**Affiliations:** 1 Institute of Pharmacy, Pharmaceutical College of Henan University, Jinming District, Kaifeng, Henan Province, China; 2 School of Pharmacy and Chemical Engineering, Zhengzhou University of Industry Technology, Xinzheng, Henan Province, China

**Keywords:** semen armeniacae amarum, COVID-19, network pharmacology, target prediction, molecular docking

## Abstract

**Background:**

Coronavirus disease 2019 (COVID-19) outbreak is progressing rapidly, and poses significant threats to public health. A number of clinical practice results showed that traditional Chinese medicine (TCM) plays a significant role for COVID-19 treatment.

**Objective:**

To explore the active components and molecular mechanism of semen *armeniacae amarum* treating COVID-19 by network pharmacology and molecular docking technology.

**Methods:**

The active components and potential targets of semen *armeniacae amarum* were retrieved from traditional Chinese medicine systems pharmacology (TCMSP) database. Coronavirus disease 2019-associated targets were collected in the GeneCards, TTD, OMIM and PubChem database. Compound target, compound-target pathway and medicine-ingredient-target disease networks were constructed by Cytoscape 3.8.0. Protein-protein interaction (PPI) networks were drawn using the STRING database and Cytoscape 3.8.0 software. David database was used for gene ontology (GO) and Kyoto Encyclopedia of Genes and Genomes (KEGG) enrichment analysis. The main active components were verified by AutoDock Vina 1.1.2 software. A lipopolysaccharide (LPS)-induced lung inflammation model in Institute of Cancer Research (ICR) mice was constructed and treated with amygdalin to confirm effects of amygdalin on lung inflammation and its underlying mechanisms by western blot analyses and immunofluorescence.

**Results:**

The network analysis revealed that nine key, active components regulated eight targets (Proto-oncogene tyrosine-protein kinase SRC (SRC), interleukin 6 (IL6), mitogen-activated protein kinase 1 (MAPK1), mitogen-activated protein kinase 3 (MAPK3), vascular endothelial growth factor A (VEGFA), epidermal growth factor receptor (EGFR), HRAS proto-oncogene (HRAS), caspase-3 (CASP3)). Gene ontology and KEGG enrichment analysis suggested that semen *armeniacae amarum* plays a role in COVID-19 by modulating 94 biological processes, 13 molecular functions, 15 cellular components and 80 potential pathways. Molecular docking indicated that amygdalin had better binding activity to key targets such as IL6, SRC, MAPK3, SARS coronavirus-2 3C-like protease (SARS-CoV-2 3CLpro) and SARS-CoV-2 angiotensin converting enzyme II (ACE2). Experimental validation revealed that the lung pathological injury and inflammatory injury were significantly increased in the model group and were improved in the amygdalin group.

**Conclusion:**

Amygdalin is a candidate compound for COVID-19 treatment by regulating IL6, SRC, MAPK1 EGFR and VEGFA to involve in PI3K-Akt signalling pathway, VEGF signalling pathway and MAPK signalling pathway. Meanwhile, amygdalin has a strong affinity for SARS-CoV-2 3CLpro and SARS-CoV-2 ACE2 and therefore prevents the virus transcription and dissemination.

## Popular scientific summary

- Amygdalin is a candidate compound for COVID-19 treatment by regulating IL6, SRC, MAPK1 EGFR and VEGFA to involve in PI3K-Akt signaling pathway, VEGF signaling pathway and MAPK signaling pathway.
- Amygdalin has a strong affinity for SARS-CoV-2 3CLpro and SARS-CoV-2 ACE2 and therefore prevents the virus transcription and dissemination.
- Amygdalin prevents LPS-induced lung inflammation.

Coronavirus disease 2019 (COVID-19), caused by a newly identified coronavirus SARS-CoV-2, has spread to more than 100 countries with 96,000 reported cases around the world, and poses significant threats to public health (1). Unfortunately, there is no medication specific to COVID-19 treatment so far (2). Traditional Chinese medicine (TCM) has been used to treat and prevent viral pneumonia for thousands of years and has been efficaciously prescribed, resulting in a lot of clinical successes (3). In the treatment of COVID-19, frequency analysis of Chinese medicine prescribing semen *armeniacae amarum* was found to be one of the herbs with the highest frequency used (4). Semen *armeniacae amarum* is the dry, ripe seed obtained from several plants of the Rosaceae family (Prunus armeniaca L. var. ansu Maxim., P. sibirica L and P. armeniaca L), and has antitussive, expectorative and antiasthmatic effects (5). Therefore, potential active ingredient in semen *armeniacae amarum* may control COVID-19 symptoms or prevent SARS-CoV-2. Network pharmacology analysis has been widely used to evaluate the interactions between proteins and molecules in biological systems, and to study the mechanisms of TCM that provide a new and powerful method for these multi-target drugs (6, 7). Therefore, this study will perform network pharmacology and animal experiments to elucidate the mechanism of semen *armeniacae amarum* on COVID-19.

## Materials and methods

### Study design

The active components and potential targets of semen *armeniacae amarum* were retrieved from traditional Chinese medicine systems pharmacology (TCMSP) database. Coronavirus disease 2019-associated targets were collected in the GeneCards, TTD, OMIM and PubChem database. Compound target, compound-target pathway and medicine-ingredient-target disease networks were constructed by Cytoscape 3.8.0. Protein-protein interaction (PPI) networks were drawn using the STRING database and Cytoscape 3.8.0 software. David database was used for gene ontology (GO) and KEGG enrichment analysis. The main active components were verified by AutoDock Vina 1.1.2 software.

### Active components and potential targets in semen armeniacae amarum

The chemical components of semen *armeniacae amarum* were identified from literature and the TCMSP (http://lsp.nwu.edu.cn/tcmsp.php/) database (8). The active compounds were mainly filtered by integrating oral bioavailability (OB) >30% and drug-likeness (DL) >0.18 (9). Then through UniProt (https://www.uniprot.org/) and Swiss Target Prediction (http://www.swisstargetprediction.ch/) database, which limits species for ‘Homo sapiens’, the correction-active ingredient obtained corresponding target genes.

### Disease-associated targets of COVID-19

In the GeneCards (https://www.genecards.org/), TTD (http://db.idrblab.net/ttd/), OMIM (https://omim.org/) and PubChem (https://pubchem.ncbi.nlm.nih.gov/) database (10), COVID-19-related genes were obtained by searching for the keywords ‘COVID-19’ and ‘coronavirus disease 2019’ and ‘novel coronavirus pneumonia’.

### Construction of medicine-ingredients-targets-disease networks

Coronavirus disease 2019-related targets and drug targets were mapped in OmicStudio (https://www.omicstudio.cn/tool/) tool to select the common targets. Then Cytoscape 3.8.0 software was used to map the medicine-ingredient-target-disease network.

### Construction of target PPI network

The common targets of semen *armeniacae amarum* and COVID-19 were imported into the string biological database (https://string-db.org/), selected with species limited to *‘Homo sapiens’* and the minimum confidence score >0.7 (11). The discrete network nodes were hidden to obtain the protein interaction data information of intersection targets. Using the network analyser function of Cytoscape 3.8.0 software to build PPI network and screen key targets (12). The node represents the protein and the edge represents the interaction between proteins. Degree of value determines the node area size; greater the node area, greater the role of proteins in the network (13). These targets with higher values of degree were identified as the candidate targets of semen *armeniacae amarum* for COVID-19.

### Gene ontology and pathway enrichment analysis

In order to elucidate the action mechanisms of semen *armeniacae amarum* on COVID-19, DAVID 6.8 (https://david.ncifcrf.gov/) (14) was used to analyse both GO biological processes and KEGG pathways (limited species: *Homo sapiens*). Then the top 20 GO enrichment analyses and the top 20 KEGG pathway enrichment analyses results were selected (*P* < 0.01). Gene set enrichment analyses were performed by using the OmicShare tools (http://www.omicshare.com/tools/), which visualised the enrichment analysis results (15). To further characterise the molecular mechanism of semen *armeniacae amarum* on COVID-19, a compound-target-pathway network was performed based on active compounds, targets and their corresponding signal pathways. In these networks, we used nodes to stand for the compounds, targets, pathways, and the edges between the two nodes represented their interaction.

### Molecular docking verification

Molecular docking was carried out between active ingredients in the medicine-ingredient-target-disease network and the top 8-degree value of the target genes in the PPI network. The mol2 file format structures of chemical compounds were obtained from the TCMSP database, and the crystal structures of core targets from the RCSB protein data bank (PDB, http://www.rcsb.org/) were collected. The three-dimensional (3D) structure of the SARS-CoV-2 3CLpro and SARS-CoV-2 angiotensin converting enzyme II (ACE2) was downloaded from the national microbiology science database (http://nmdc.cn/#/resource/detail? No=NMDCS0000004, No=NMDCS0000001). The molecular structure documents of the main active components and key target genes of semen *armeniacae amarum* were converted into one format which stores the atomic coordinates, partial charges and Autodock atom types, for both the receptor and the ligand in Autodock tools 1.5.6 software, and molecular docking was performed by using Autodock Vina 1.1.2 software. PyMOL 2.3.2 software was used to visually analyse the results with higher docking scores (16).

#### Materials

Amygdalin (purity: High Performance Liquid Chromatography (HPLC) ≥ 98%, DST200710 004) was purchased from Lemeitian Pharmaceutical Technology Co., Ltd. (Chengdu, China). Lipopolysaccharide (LPS) was purchased from Sigma Chemical Co. (St. Louis, MO, United States). Reactive Oxygen Species Assay Kit (ROS, DCFH-DA) were obtained from Beijing Solarbio Technology Co., Ltd. (Beijing, China). Antibodies used herein including anti-IL6, anti-TNF-α, anti-p-AKT, anti-AKT, anti-SRC, anti-p-SRC, anti-VEGFA, anti-MAPK1, anti-EGFR, anti-IL-1β, anti-TGF-β1, β-actin, fluorescein isothiocyanate (FITC)-conjugated goat anti-mouse IgG and Horseradish Peroxidase (HRP)-conjugated goat anti-mouse IgG polyclonal antibody were obtained from R&D Systems (Minnesota, USA).

#### Animals and treatment

Eight-week old female ICR mice were obtained from Henan Provincial Medical Laboratory Animal Center. All mice were housed in individual ventilated cages and fed standard rodent chow and water. All animal procedures were approved by the Animal Experimentation Ethics Committee of Henan University (permission number HUSAM 2016-288), and all procedures were performed in strict accordance with the Guide for the Care and Use of Laboratory Animals and the Regulation of Animal Protection Committee to minimise suffering and injury. Animals were euthanised via carbon dioxide overdose based on the experimental requirement. Standard rodent chow was purchased from Henan Provincial Medical Laboratory Animal Center (Zhengzhou, China), License No. SCXK (YU) 2015-0005, Certificate No. 41000100002406.

The animals were divided into five groups (*n* = 10/group): control group, LPS group, LPS + amygdalin (0.5, 1, 2 mg/kg) group. The LPS-induced lung inflammation model was established in mice that received an instilling intratracheal of LPS (1 mg/kg) once weekly for three weeks. Following the first LPS instillation, the treated mice received amygdalin intraperitoneally (i.p.) once daily for 3 weeks. Food and water were provided *ad libitum* during the study. The health of the mice was monitored daily, and body weights were measured weekly. Lung function was analysed weekly by tidal volume (TV) using the animal respiratory metabolic measurement system (Sable Systems International, United States).

#### Histopathological evaluation

Three weeks following drug delivery, mice were anesthetised with pentobarbital sodium (90 mg/kg), a part of each lung was preserved in 10% buffered formalin and routinely embedded in paraffin. Lung sections were stained with haematoxylin and eosin (H&E), the pathological score was determined as previously reported (17).

#### Lung W/D ratio

After the mice were killed humanely, lung tissues were collected and weighed immediately. Then the tissues were heated at 80°C for 48 h to obtain the dry weight. The lung wet/dry (W/D) ratio was calculated by dividing the wet weight by the dry weight (18).

#### ROS assay

ROS were determined by enzyme-linked immunosorbent assay (ELISA) kits, as per the manufacturer’s instructions (Model: F-4600 FL Spectrophotometer).

#### Immunofluorescence staining

Lung tissues sections were treated with blocked 5% bovine serum albumin (BSA) for 30 min at room temperature and incubated with anti-IL-6, anti-TGF-β1, anti-TNF-α, anti-IL-1β, anti-VEGFA, anti-MAPK1, anti-EGFR, and secondary antibody (FITC-labeled goat anti-rabbit IgG). Then the sections were fixed with anti-fluorescence quencher, observed and photographed under the fluorescence microscope. Fluorescence intensity was quantified using ImageJ software (NIH, Bethesda, MD, USA).

#### Western blot analysis

Protein extracts were separated using sodium dodecyl sulfate-polyacrylamide gel electrophoresis and then transferred to polyvinylidene fluoride membranes (Millipore, Germany). Protein expression was detected by western blot analysis. Antibodies used herein including anti-IL-6, anti-TNF-α, anti-p-AKT, anti-AKT, anti-SRC, anti-p-SRC, anti-VEGFA, anti-MAPK1, anti-IL-1β, anti-EGFR and β-actin were obtained from R&D Systems. Band density was quantified using ImageJ software and normalised to the corresponding control group.

#### Statistical analyses

The data was statistically analysed using GraphPad Prism, Version 5.0 (San Diego, CA, USA) and presented as the mean ± SD. The differences between the two groups were evaluated using a *t*-test. A *P*-value of less than 0.05 was considered statistically significant.

## Results

### The flow chart of the whole analysis for this study

**Fig. 1:**
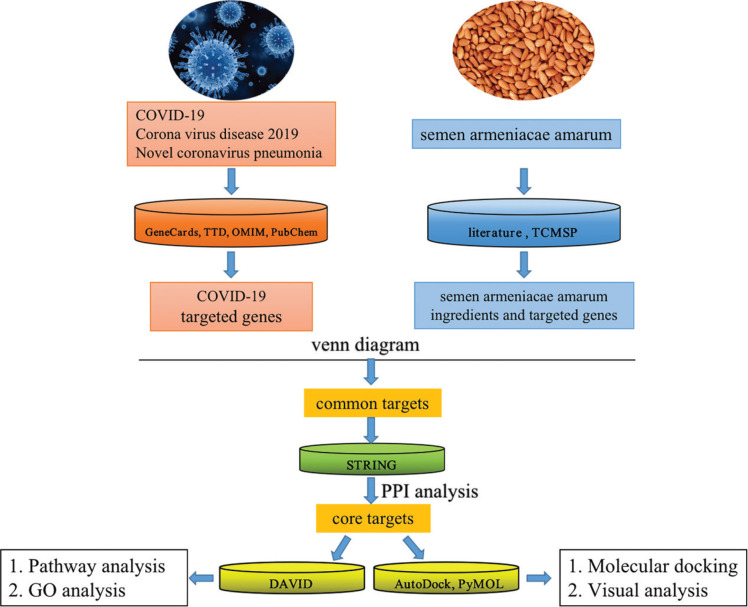
Flow chart of the entire analysis for this study.

The steps used in the entire analysis performed in this study are detailed in Fig. 1.

### *Active components and potential targets in semen armeniacae* amarum

**Fig. 2:**
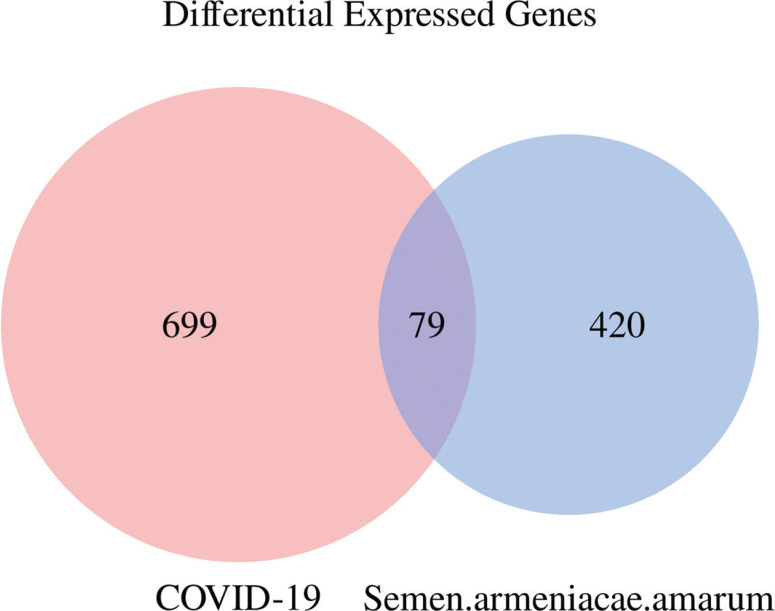
Component-target network: Network with 508 nodes and 1092 edges linking nine compounds in semen *armeniacae amarum* and 499 target genes. The red nodes represent the compounds, the green and yellow nodes represent the targets and those compounds that are linked with the corresponding targets. Degree of value determines the node area size, greater the node area, greater the role of proteins in the network.

Through the literature and TCMSP database search, 85 ingredients were obtained. Among them, amygdalin is a major effective component of semen *armeniacae amarum* (19). Nine active compounds were ultimately chosen for further investigation. From the Swiss Target Prediction database and TCMSP database results, we obtained potential targets for all nine active compounds (Table 1). A Total of 499 targets were identified for nine compounds of semen *armeniacae amarum*, deleting duplicate targets of the same compound. Information about the target of active compounds in semen *armeniacae amarum* is shown in Fig. 2.

**Table 1:** Information on nine active ingredients of semen *armeniacae amarum* and the number of corresponding targets

MOL ID	Molecule name	Oral bioavailability/%	Drug-likeness	Target number
MOL000449	Stigmasterol	43.82	0.75	145
MOL000359	Sitosterol	36.91	0.75	106
MOL000953	Cholesterol (CLR)	37.87	0.67	121
MOL002372	(6Z,10E,14E,18E)-2,6,10,15,19,23- hexamethyltetracosa-2,6,10,14,18,22-hexaene	33.54	0.42	108
MOL010921	Oestrone	53.56	0.31	149
MOL010922	Diisooctyl succinate	31.61	0.23	119
MOL002211	11,14-eicosadienoic acid	39.99	0.20	114
MOL005030	Gondoic acid	30.70	0.19	108
MOL001320	Amygdalin	4.415	0.61	122

### COVID-19-related targets and medicine-ingredient-target-disease networks

**Fig. 3:**
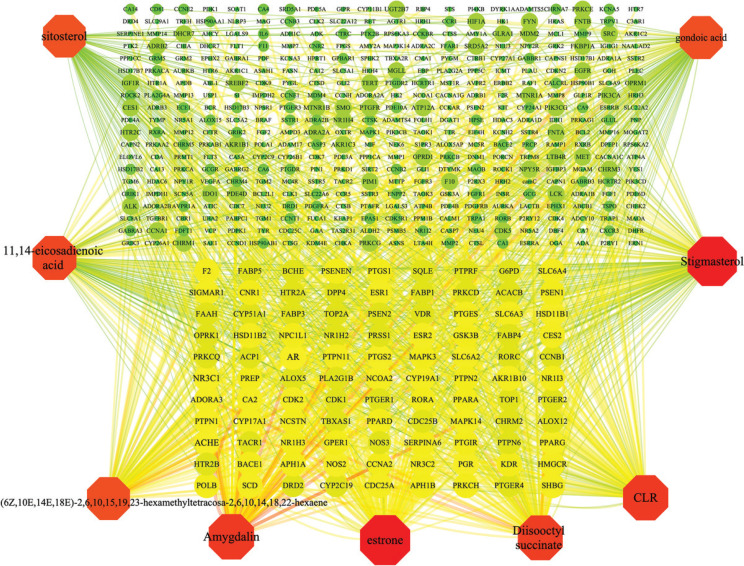
Venn diagram of semen *armeniacae amarum*-COVID-19 intersection targets. The 778 targets of COVID-19 were mapped to the 499 targets of semen *armeniacae amarum* to screen out the 79 common targets.

**Fig. 4:**
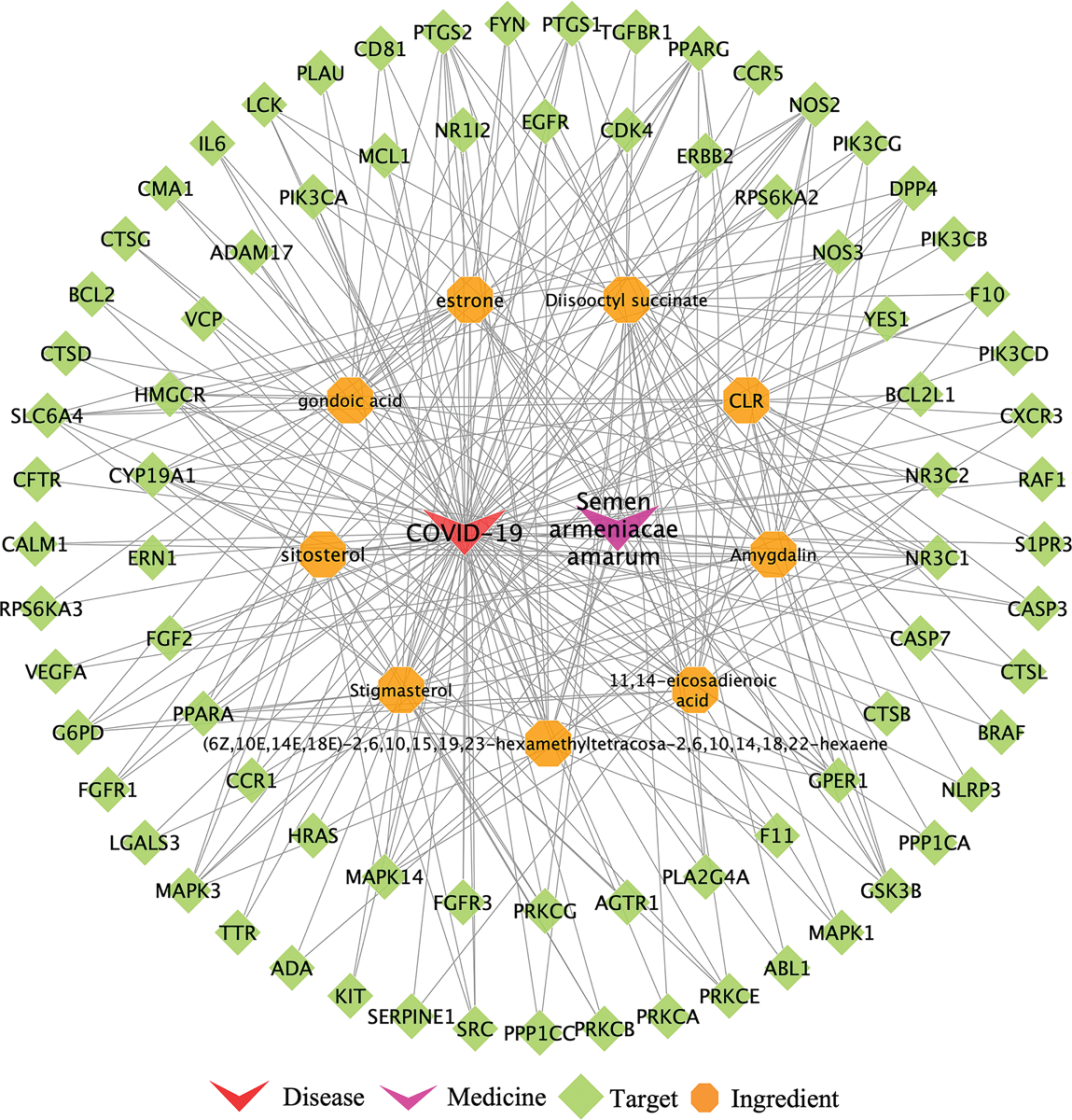
Medicine-ingredient-target-disease network: The medicine-ingredient-target-disease network consists of 90 nodes and 280 edges. The red arrows indicate disease. The purple arrows represent medicine. The green diamonds represent the targets. The orange octagons represent the ingredients.

Through GeneCards, TTD, OMIM and PubChem database, 778 COVID-19 disease targets were obtained. Then 499 drug targets and 778 COVID-19-related targets were mapped using a Venn diagram in OmicStudio tool, 79 common targets were obtained (Fig. 3). The medicine-ingredient-target-disease network consists of 90 nodes and 280 edges (Fig. 4).

### Target PPI network

**Fig. 5:**
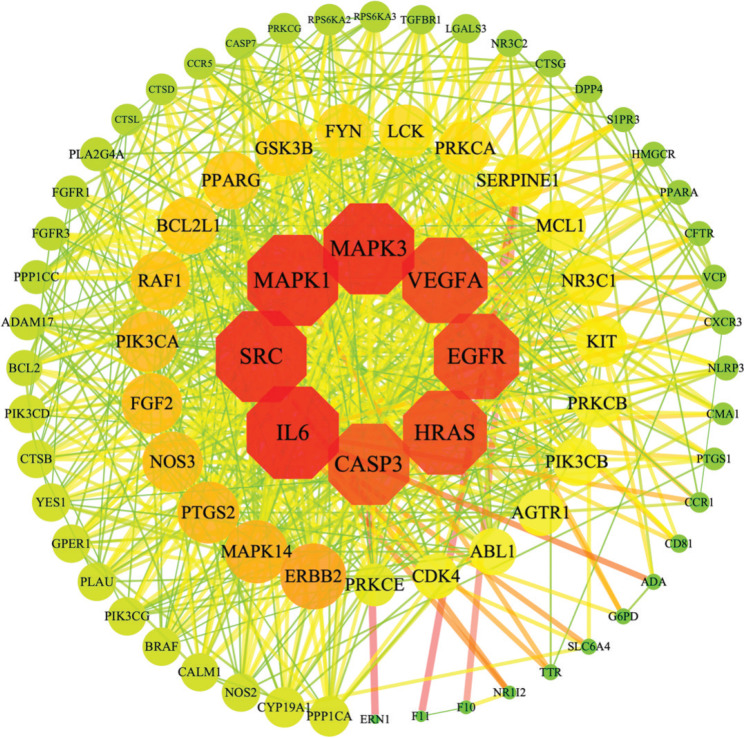
Semen *armeniacae amarum*-COVID-19 intersection target PPI network. Red octagon represents core targets (IL6, SRC, MAPK3, MAPK1, EGFR, VEGFA, HRAS, CASP3).

In the Cytoscape 3.8.0 software, the PPI network of the 79 targets was established (Fig. 5). Protein–protein interactions network analysis results show that PPI network contain 79 nodes and 691 edges, and the average degree of nodes is 17.5. Degree of value determines the node area size, greater the node area, greater the role of proteins in the network. These eight targets with higher values of ‘Degree’ (above twofold of the median value) were identified as the candidate semen *armeniacae amarum* targets for COVID-19, including IL6(51), SRC(50), MAPK3(50), MAPK1(50), EGFR(46), VEGFA(46), HRAS(45) and CASP3(43).

### Gene Ontology biological process and KEGG pathway enrichment analysis

**Fig. 6:**
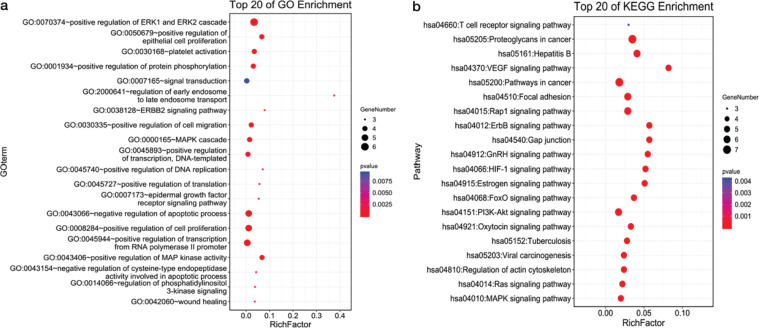
Gene Ontology biological process and KEGG pathway enrichment analysis network. Bubble chart of 20 biological processes and 20 signalling pathways related to occurrence and development of COVID-19. (a) The top 20 GO enrichment analysis results were selected (*P* < 0.01). (b) The top 20 KEGG pathway enrichment analysis results were selected (*P* < 0.01).

Eight predicted targets were imported in the DAVID 6.8 database to obtain 94 GO biological processes and 80 KEGG pathways. Gene Ontology biological processes are mainly involved in positive regulation of ERK1 and ERK2 cascade, positive regulation of cell proliferation, negative regulation of apoptotic process, positive regulation of MAP kinase activity, etc. The main pathways are PI3K-Akt signalling pathway, VEGF signalling pathway and MAPK signalling pathway. Gene Ontology biological processes and KEGG pathway were visualised in the enrichment analysis results via the OmicShare tools (Fig. 6a, b).

### Component-target-pathway network

**Fig. 7:**
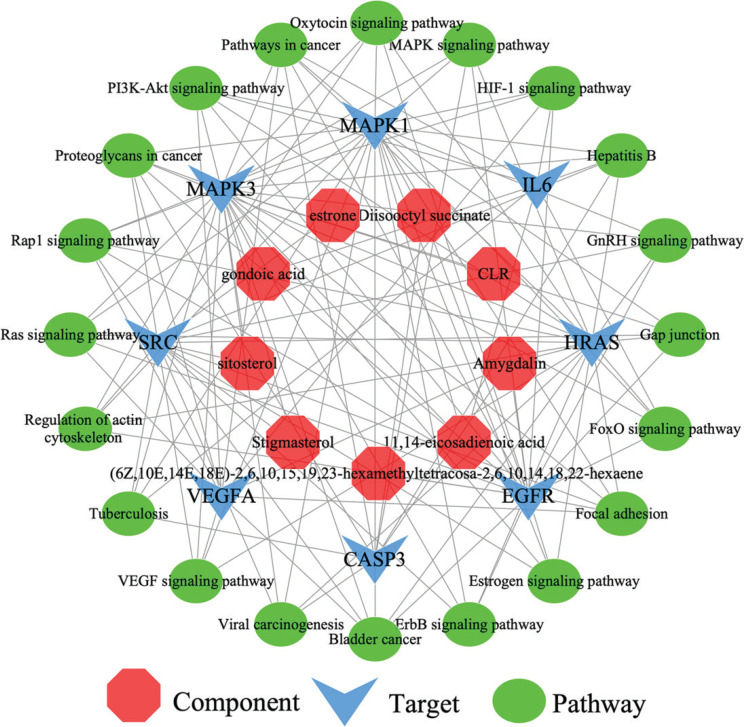
Component-target-pathway network. The component-target-pathway network consists of 37 nodes and 128 edges. The red arrows indicate disease. The red octagons represent the components. The blue arrows represent the targets. The green ovals represent the pathways.

Twenty signal pathways were selected in the treatment of COVID-19, constructing the component-target-pathway network (Fig. 7). This network interacts with 37 nodes and 128 interaction edges.

### Molecular docking verification

**Fig. 8:**
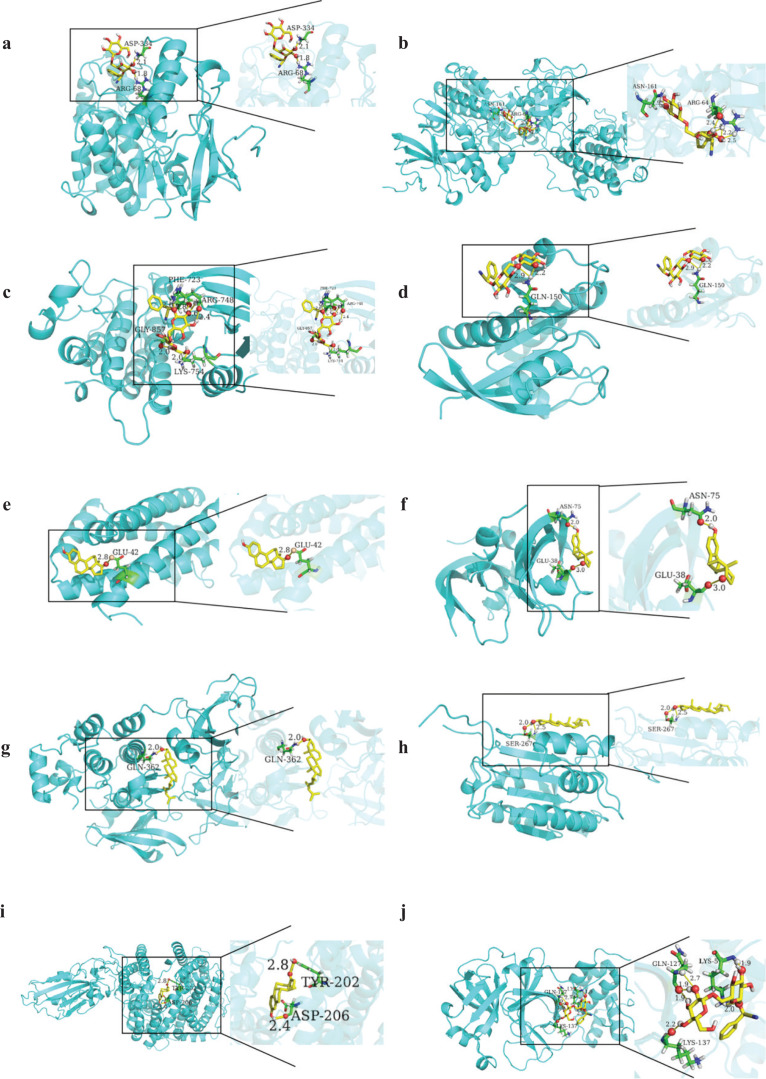
Pattern diagram of molecular docking. (a) Amygdalin-MAPK1; (b) Amygdalin-MAPK3; (c) Amygdalin-EGFR; (d) Amygdalin-HRAS; (e) Estrone-IL6; (f) Estrone-VEGFA; (g) Stigmasterol-SRC; (h) Sitosterol-CASP3; (i) Estrone-SARS-CoV-2 ACE2; (j) Amygdalin-SARS-CoV-2 3CLpro.

AutoDock Vina 1.1.2 software was used to dock the semen *armeniacae amarum* active components with the core targets of IL6, SRC, MAPK3, MAPK1, EGFR, VEGFA, HRAS, CASP3, SARS-CoV-2 ACE2, SARS-CoV-2 3CLpro, respectively, (Table 2). Binding energy less than 0 indicates spontaneous binding of ligand and receptor. More stable the binding conformation, lower the binding energy and greater the possibility of action. It is generally believed that a binding energy greater than −4.25 kcal/mol indicates a good binding activity between the ligand small molecule, and the receptor-protein selected docking results in better data (20). Results showed that amygdalin, oestrone, stigmasterol, sitosterol and cholesterol (CLR) showed strong affinity for core targets. Among them, amygdalin showed strong affinity for SARS-CoV-2 ACE2 and SARS-CoV-2 3CLpro. The better docking results were selected for molecular docking visualisation analysis with PyMOL 2.3.2 software (Fig. 8). The dotted line in the figure is hydrogen bond, and the value is the bond length.

**Table 2:** Molecular docking of main active ingredients of semen *armeniacae amarum* and corresponding targets

Target name	Protein data bank ID	Compound	Energy (kcal/mol)
IL6	1alu	Stigmasterol	−5.77
IL6	1alu	Sitosterol	−5.53
IL6	1alu	Oestrone	−6.45
IL6	1alu	Cholesterol	−6.07
IL6	1alu	Amygdalin	−6.37
SRC	1fmk	Stigmasterol	−6.33
SRC	1fmk	Sitosterol	−4.71
SRC	1fmk	Oestrone	−5.99
SRC	1fmk	Cholesterol	−5.54
SRC	1fmk	Amygdalin	−6.04
MAPK1	4zzn	Stigmasterol	−4.41
MAPK1	4zzn	Sitosterol	−4.57
MAPK1	4zzn	Cholesterol	−4.28
MAPK1	4zzn	Oestrone	−5.46
MAPK1	4zzn	Amygdalin	−7.92
MAPK1	4zzn	Gondoic acid	−5.14
MAPK3	4qtb	Stigmasterol	−5.14
MAPK3	4qtb	Sitosterol	−4.39
MAPK3	4qtb	Cholesterol	−5.55
MAPK3	4qtb	Oestrone	−5.09
MAPK3	4qtb	Amygdalin	−8.91
MAPK3	4qtb	Diisooctyl succinate	−6.12
VEGFA	1mkk	Stigmasterol	−5.36
VEGFA	1mkk	Sitosterol	−4.99
VEGFA	1mkk	Oestrone	−6.07
VEGFA	1mkk	Cholesterol	−5.27
VEGFA	1mkk	Amygdalin	−4.62
EGFR	5ug9	Stigmasterol	−7.21
EGFR	5ug9	Sitosterol	−4.59
EGFR	5ug9	Cholesterol	−4.71
EGFR	5ug9	Oestrone	−6.37
EGFR	5ug9	Amygdalin	−7.75
EGFR	5ug9	11,14-eicosadienoic acid	−5.62
HRAS	2ce2	Stigmasterol	−5.86
HRAS	2ce2	Sitosterol	−4.83
HRAS	2ce2	Cholesterol	−5.56
HRAS	2ce2	Oestrone	−5.69
HRAS	2ce2	Amygdalin	−8.80
HRAS	2ce2	Gondoic acid	−6.32
CASP3	2j32	Stigmasterol	−5.53
CASP3	2j32	Sitosterol	−6.33
CASP3	2j32	Oestrone	−5.99
CASP3	2j32	Cholesterol	−5.10
CASP3	2j32	Amygdalin	−5.04
SARS-CoV-2 ACE2	6LZG	Stigmasterol	−5.44
SARS-CoV-2 ACE2	6LZG	Sitosterol	−5.42
SARS-CoV-2 ACE2	6LZG	Cholesterol	−6.27
SARS-CoV-2 ACE2	6LZG	Oestrone	−6.37
SARS-CoV-2 ACE2	6LZG	Amygdalin	−5.36
SARS-CoV-2 3CLpro	6LU7	Stigmasterol	−6.47
SARS-CoV-2 3CLpro	6LU7	Sitosterol	−5.78
SARS-CoV-2 3CLpro	6LU7	Oestrone	−6.29
SARS-CoV-2 3CLpro	6LU7	Amygdalin	−7.44
SARS-CoV-2 3CLpro	6LU7	Cholesterol	−6.18

Oestrone combined with IL6 to form hydrogen bond with the active site, GLU-42 amino acids bind with VEGFA and forms hydrogen bonds with the two amino acids ASN-75 and GLU-38 near the active site. Stigmasterol binds to SRC and forms hydrogen bond with the active site of Gln-362 amino acids. Amygdalin binds to MAPK1 and forms hydrogen bonds with the two amino acids near the active site of ASP-334, and ARG-68 binds to MAPK3 and forms hydrogen bonds with the two amino acids of ASN-161 and ARG-64 near the active site, EGFR binds to and interacts with the four amino acids PHE-723, ARG-748, LYS-754, GLY-857 near the active site to form hydrogen bonds, HRAS and binds to and forms hydrogen bond interactions with the active site of GLN-150 amino acids. Sitosterol binds to CASP3 and forms hydrogen bond interactions with the active site of SER-267 amino acids. Besides, oestrone binds to SARS-CoV-2 ACE2, and forms a hydrogen bond interaction with the two amino acids TYR-202, ASP-206 near the active site. Amygdalin binds to SARS-CoV-2 3CLpro and interacts with the three amino acids GLN-127, LYS-5, LYS-137 near the active site to form hydrogen bonds.

#### Amygdalin prevents LPS-induced lung inflammation

**Fig. 9:**
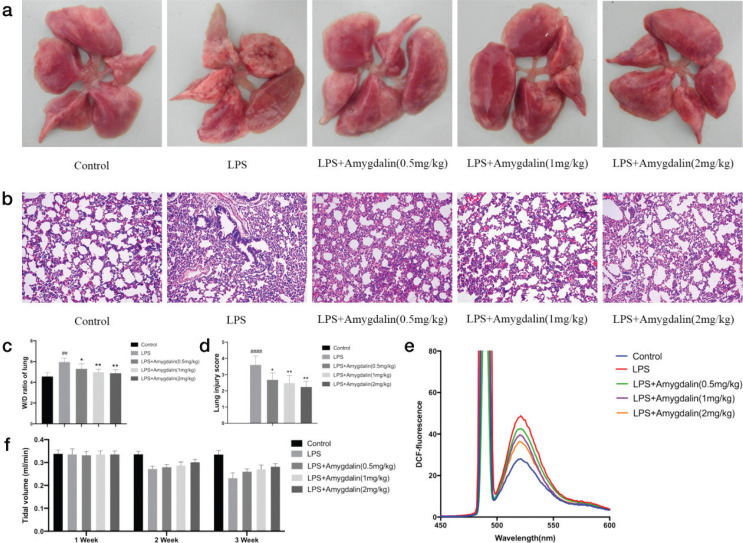
Effects of amygdalin on histopathological changes in lung tissues of LPS-induced lung-inflammatory mice. (a) The whole lung in naked eye (*n* = 5). (b) HE staining of lung tissues (*n* = 5). (c) W/D ratio of lung tissues (*n* = 5). (d) Lung injury score (*n* = 5). (e) ROS detected by DCFH-DA (*n* = 5). (f) Tidal volume (TV) in lung function (*n* = 5). ^##^*P* < 0.01, ^####^*P* < 0.0001 versus Control; ^*^P < 0.05, ^**^P < 0.01 versus LPS group.

The TV was used for detecting lung function. As shown in Fig. 9f, TV was significantly decreased in the LPS group as compared with the control group, but increased in amygdalin group (TV in the lung function decreased more than 30% among LPS mice). The lung tissues of LPS group exhibited notably pathologic changes, including alveolar disarray, increased alveolar wall thickness and inflammatory cell infiltration. Compared to the control group, amygdalin treatment significantly alleviated LPS-induced lung pathologic changes (Fig. 9a–d). In addition, ROS was also significantly increased in LPS group as compared with the control group, but decreased in amygdalin group (Fig. 9e).

#### Amygdalin suppresses lung inflammation-related targets induced by LPS

**Fig. 10:**
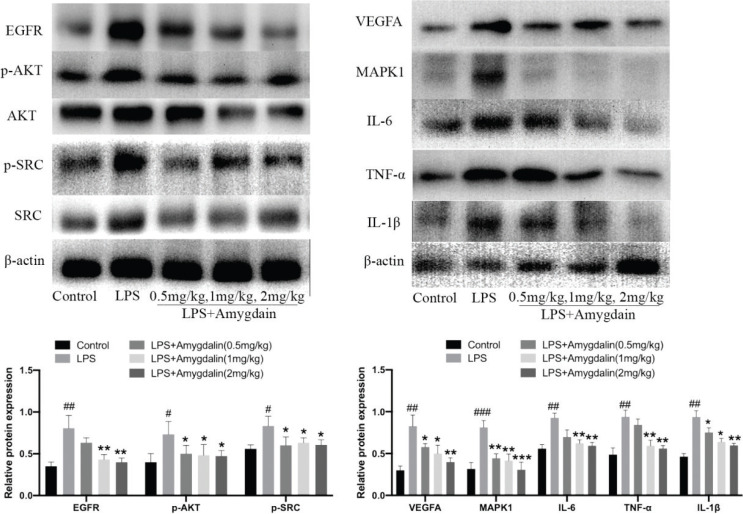
Amygdalin suppresses lung inflammation-related targets induced by LPS. Core target-associated markers examined by western blot (*n* = 3). The data presents mean ± SD, the experiments were repeated three times, and statistical significance was determined by a *t*-test. ^#^*P* < 0.05, ^##^*P* < 0.01, ^###^*P* < 0.001 versus Control; ^*^P < 0.05, ^**^P < 0.01, ^***^P < 0.001 versus LPS group.

**Fig. 11:**
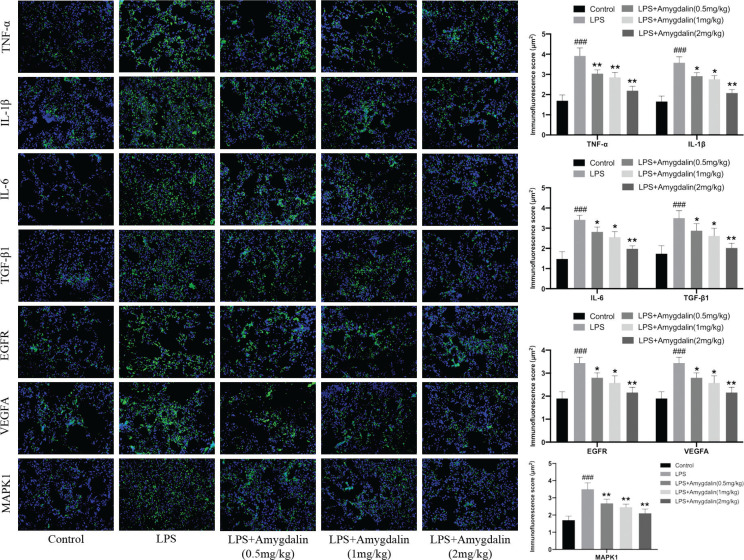
Amygdalin suppresses lung inflammation-related targets induced by LPS. Core targets-associated markers examined by immunofluorescence (*n* = 3). Immunofluorescence score of lung inflammation-related targets (*n* = 3). The data presents mean ± SD, the experiments were repeated three times, and statistical significance was determined by a *t*-test. ^#^*P* < 0.05, ^##^*P* < 0.01, ^###^*P* < 0.001 versus Control; ^*^P < 0.05, ^**^P < 0.01, ^***^P < 0.001 versus LPS group.

Western blot analyses were performed to evaluate related targets involved in the therapeutic effects of amygdalin on LPS-induced lung inflammation. Results showed that the expression of inflammation-related target proteins (such as IL-6, MAPK1, TNF-α, p-AKT, p-SRC, IL-1β, VEGFA and EGFR) obviously increased in the LPS group and amygdalin could decrease this inflammation (Fig. 10). Immunofluorescence further confirms these results (Fig. 11).

## Discussion

Coronavirus disease 2019 is a new clinical syndrome characterised by respiratory symptoms with varying degrees of severity, from mild respiratory disease to severe interstitial pneumonia (21). Viral pneumonia can be defined as abnormal alveolar gas exchange and inflammation of lung parenchyma via western medicine (22). While in the theoretical system of TCM through the skin, mouth and nose, the weak lungs were invaded by an external pathogen causing lung gas obstruction and stagnation (23). The main pathological products of COVID-19 are heat, phlegm and blood stasis, manifested by cough, fever, wheezing, dyspnea, etc. (24). Regulating the lung gas resolves phlegm and relieves cough and dyspnea, deemed to be the principle behind the basic treatment. Semen *armeniacae amarum* although tasting bitter, functions by reducing lung gas; has anti-cough, anti-asthma, anti-inflammatory, analgesic, anti-oxidation, anti-tumor, anti-thrombus and other pharmacological effects (25). Although semen *armeniacae amarum* is widely used in respiratory diseases such as pneumonia, bronchitis and asthma (26), its active compounds are not clear in clinical and pharmacological studies; the specific targets have not fully been identified and the mechanism has not been elaborated. Network pharmacology is a powerful tool for elucidating complex and holistic mechanisms of TCM. It can illustrate the intricate interactions among drugs, diseases, proteins and genes from a network perspective (27–29). Here, we used network pharmacology and animal experiments to elucidate the mechanisms of multiple target components in semen *armeniacae amarum*.

In this study, we screened out nine main active components of semen *armeniacae amarum*. Among them, stigmasterol performs anti-inflammatory activity, and suppresses airway inflammation by inhibiting allergen-induced immunoglobulin E-mediated responses (30, 31). Sitosterol has antioxidant activity (32). Amygdalin has anti-cough, anti-inflammatory, antibiotic, anti-tumor and other pharmacological effects (33, 34). In addition, a lot of research shows amygdalin also possesses antitussive activities via inhibiting the central cough centre when it is bio-transformed into cyanide (35). Therefore, these compounds are closely associated with COVID-19 treatment.

Medicine-ingredient-target-disease network and PPI network analysis showed that IL6, SRC, MAPK1, MAPK3, VEGFA, EGFR, HRAS and CASP3 may be the core targets of semen *armeniacae amarum* treating COVID-19. These core target proteins are mainly related to inflammation and immunoregulation (36). Kyoto Encyclopedia of Genes and Genomes enrichment analysis showed that the core targets were mainly concentrated in PI3K-Akt signalling pathway, VEGF signalling pathway, Rap 1 signalling pathway and MAPK signalling pathway. The PI3K-AKT signalling pathway plays a role in inflammatory response in the lungs and airways by regulating the release of inflammatory transmitters and the activation of inflammatory response cells (37). Vascular endothelial growth factor (VEGF) signalling pathway was an important pathway in inflammatory response, in which related factor receptors can induce apoptosis (38). Ras-related protein 1 (Rap 1) is a negative regulator of mitochondrial ROS production, and the Rap 1 signalling pathway regulates ROS production (39). The MAPK pathway is a key mediator of inflammation implicated in injury of lungs (40). Therefore, semen *armeniacae amarum* may play anti-inflammatory, antioxidant and immunological roles by regulating PI3K-Akt signalling pathway, VEGF signalling pathway and MAPK signalling pathway. In order to obtain more explainations, we searched the GWAS database, however, there was no relevant data. We also wanted to find relevant data on the Global initiative on sharing all influenza data (GISAID) platform but regretfully, we were unable to register successfully.

Molecular docking is a powerful tool to study and provide a proper understanding of receptor-ligand interactions (41). The study showed that SARS-CoV-2 fastened to ACE2 receptors with greater affinity than SARS-CoV (42), and the SARS-CoV-2 with ACE2 combination was the main cause of COVID-19. In addition, the viral 3-chymotrypsin-like cysteine protease (3CLpro) enzyme inhibits coronavirus replication and is critical to its life cycle (43). Therefore, SARS-CoV-2, ACE2 and SARS-CoV-2 3CLpro were regarded as receptors in molecular docking. Nine main active components were used for molecular docking with core targets, SARS-CoV-2 3CLpro and SARS-CoV-2 ACE2, respectively. Results showed that amygdalin, oestrone, stigmasterol, sitosterol and CLR showed strong affinity for core targets. Among them, amygdalin showed strong affinity for SARS-CoV-2 ACE2, and SARS-CoV-2 3CLpro. Therefore, amygdalin might play an important role in the treatment of COVID-19.

Studies have shown that acute lung injury (ALI) and acute respiratory distress syndrome (ARDS) with cytokine storms might be the main cause of death due to COVID-19 (44). Many inflammatory cytokines (Interferon-alpha (IFN-α), interleukin-1beta (IL-1β), interleukin 6 (IL6), interleukin 12 (IL-12), tumor necrosis factor-alpha (TNF-α), and transforming growth factor-beta (TGFβ) and chemokines were detected in COVID-19 patients (45). Among them, IL-6 is an important factor found elevated during the pathology of COVID-19 with a cytokine storm (46). Thus, we validated the effects of amygdalin on lung inflammation induced by LPS. The results presented a reduction of TV and severe lung injury under LPS stimulation, while treatment with amygdalin prominently improved this condition. Additionally, western blot and immunofluorescence analyses revealed that the protein expression of inflammation-related targets in lung tissue was significantly increased in the LPS group and was prevented in the amygdalin group. Therefore, amygdalin is a candidate compound for COVID-19 treatment by regulating IL6, SRC, MAPK1, MAPK3, VEGFA and EGFR.

## Conclusion

In summary, amygdalin is a candidate compound for COVID-19 treatment by regulating IL6, SRC, MAPK1, EGFR and VEGFA by way of the PI3K-Akt signalling pathway, VEGF signalling pathway and MAPK signalling pathway. It was found that amygdalin has a strong affinity for SARS-CoV-2 3CLpro and SARS-CoV-2 ACE2, there by preventing the virus transcription and dissemination. The strategy of integrating classical pharmacology with systems pharmacology analysis has the potential to provide a better strategy for the better understanding of TCM mechanism.
